# Patient and Disease–Specific Induced Pluripotent Stem Cells for Discovery of Personalized Cardiovascular Drugs and Therapeutics

**DOI:** 10.1124/pr.116.013003

**Published:** 2020-01

**Authors:** David T. Paik, Mark Chandy, Joseph C. Wu

**Affiliations:** Stanford Cardiovascular Institute, Stanford University, Stanford, California

## Abstract

Human induced pluripotent stem cells (iPSCs) have emerged as an effective platform for regenerative therapy, disease modeling, and drug discovery. iPSCs allow for the production of limitless supply of patient-specific somatic cells that enable advancement in cardiovascular precision medicine. Over the past decade, researchers have developed protocols to differentiate iPSCs to multiple cardiovascular lineages, as well as to enhance the maturity and functionality of these cells. Despite significant advances, drug therapy and discovery for cardiovascular disease have lagged behind other fields such as oncology. We speculate that this paucity of drug discovery is due to a previous lack of efficient, reproducible, and translational model systems. Notably, existing drug discovery and testing platforms rely on animal studies and clinical trials, but investigations in animal models have inherent limitations due to interspecies differences. Moreover, clinical trials are inherently flawed by assuming that all individuals with a disease will respond identically to a therapy, ignoring the genetic and epigenomic variations that define our individuality. With ever-improving differentiation and phenotyping methods, patient-specific iPSC-derived cardiovascular cells allow unprecedented opportunities to discover new drug targets and screen compounds for cardiovascular disease. Imbued with the genetic information of an individual, iPSCs will vastly improve our ability to test drugs efficiently, as well as tailor and titrate drug therapy for each patient.

## I. Introduction

**Fig. 1.**
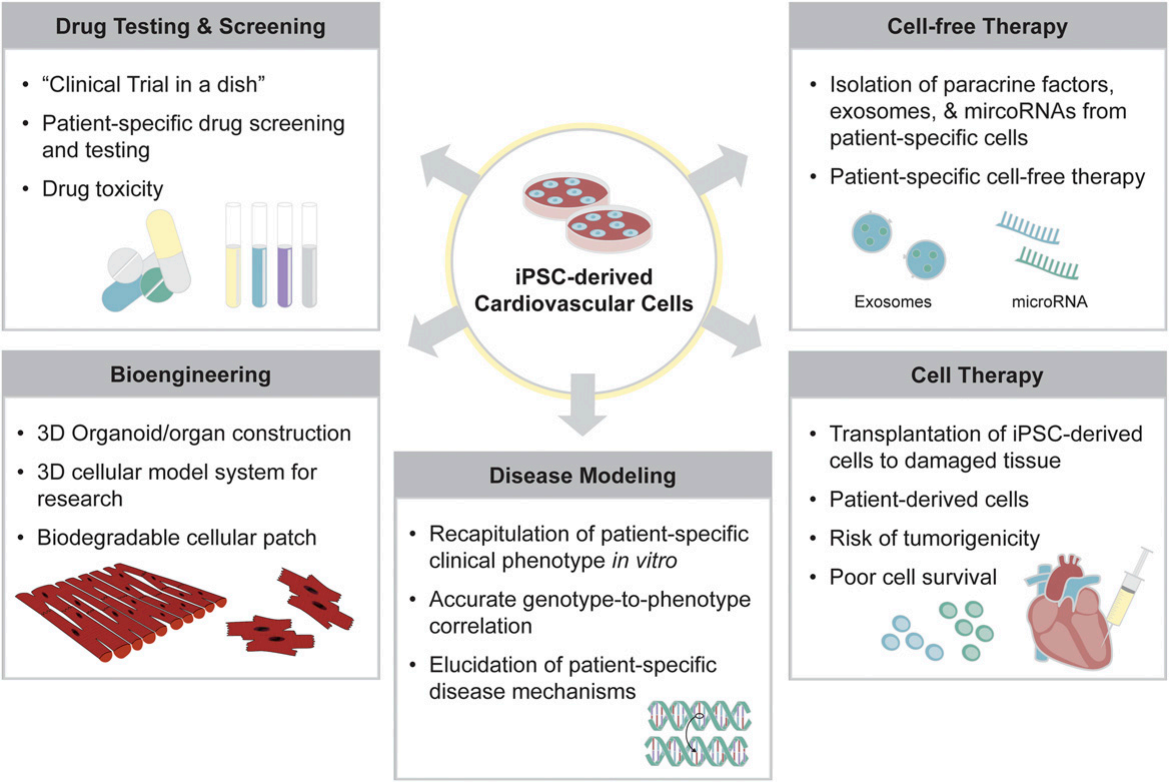
Applications of human iPSCs for precision medicine. Human iPSCs are differentiated to functional cardiovascular cells, providing an effective platform for patient-specific disease modeling, cell-based therapy, cell-free therapy, drug testing and screening, and bioengineered tissue construction. First, iPSC-derived cardiovascular cells can recapitulate patient-specific clinical phenotype in vitro, resulting in accurate genotype-to-phenotype correlation. iPSC-derived cells allow elucidation of patient-specific disease mechanisms, enabling drug screening and toxicity testing that are unique to the individual’s genetic and epigenetic makeup. iPSC-derived cells are also a source of cell-based therapy, allowing a patient’s own cells to be transplanted to the damaged tissue. In addition, exosomes and microRNAs secreted from patient-specific iPSC-derived cells allow them to be used for cell-free therapeutic purposes. Lastly, iPSC-derived cardiovascular cells can be engineered to create three-dimensional organoids or organ-like mimics of the heart or the blood vessels for advanced disease modeling. Overall, the risk of tumorigenicity and poor cell survival rate remain as challenges to be addressed.

The groundbreaking discovery by Shinya Yamanaka and colleagues that a set of four transcription factors (Oct4/Sox2/c-Myc/Klf4) can induce reprogramming of somatic cells to induced pluripotent stem cells (iPSCs) has revolutionized the field of biomedical research, providing an accessible, versatile, and adaptable platform for precision medicine (Takahashi et al. 2007). iPSCs generated from an individual can subsequently be differentiated to a wide variety of functional somatic cells, which can be used for cell or cell-free therapy for regenerative medicine, in vitro patient-specific disease modeling, drug testing, toxicity screening, and three-dimensional organ/organoid construction (Shi et al., 2017) (Fig. 1). In this review, we will examine in depth the current state and the future applications of iPSC technology to advance cardiovascular medicine and to improve drug discovery methodologies.

Drug discovery requires years of preclinical research. After a compound is synthesized, it must be rigorously tested in preclinical studies (Dahlin et al., 2015). Current models include primary cell culture and animal models, the aim of which is to demonstrate proof of principle that the drug under study is efficacious with minimal side effects. Once this proof of principle is established, the drug is eligible for clinical testing. The Food and Drug Administration (FDA) uses properly designed, double-blinded, multicenter trials to test new drugs. As a result, after years of research and testing, only a small fraction of drugs is introduced to the market.

Although animal models and primary cell lines are the most common methods for establishing efficacy and safety in preclinical drug trials, there are significant problems with each approach. Animal model systems are inherently limited due to fundamental species differences in physiology, reproducibility, ethical concerns, and a poor correlation with human clinical trial data (Begley and Ellis, 2012; Libby, 2015). For example, mouse hearts beat at 500 beats per minute, whereas human hearts normally range between 60 and 100 beats per minute, limiting the utility of mice to study the effects of anti-arrhythmic drugs. Animal model studies are also difficult to reproduce (Liao and Zhang, 2008). Primary cells extracted from human donors more directly reflect human physiology and pathology than animal models, but the former are difficult to extract and maintain. For example, human coronary endothelial cells must be extracted from the coronary arteries of human donors, a highly invasive procedure that yields few cells that cannot be sufficiently expanded in culture. As a result, coronary endothelial cells are often pooled, eliminating any chance of ascertaining patient specificity. Pools may also include cells isolated from both healthy and diseased subjects, which can muddle results. Consequently, it is imperative that we generate low-cost, quick procedures to discover test drugs, and that we identify and tailor drugs designed specific to the individual patient (Dugger et al., 2018).

As an alternative to animal models and primary cells, iPSC technology has triggered a paradigm shift in the drug discovery and clinical trial landscapes. iPSCs circumvent many of the problems associated with animal and primary cell models and allow for the mass production of disease- and patient-specific functional somatic cells (Shi et al., 2017). In this study, we will discuss the use of patient-specific iPSCs for generation of cardiovascular cell types, the strategies for drug discovery using iPSCs, and the use of iPSCs in precision cardiovascular medicine.

## II. Induced Pluripotent Stem Cells: A Platform for Precision Medicine

The generation of cardiovascular cells derived from patients allows individualized disease modeling, drug screening, and drug testing. Currently, there are two approaches to generate patient-specific pluripotent stem cells from which cardiovascular cells can be derived: 1) generation of iPSCs by ectopic overexpression of the Yamanaka transcription factors, and 2) somatic cell nuclear transfer–derived embryonic stem cells using enucleated human oocytes, a more technically challenging and ethically fraught procedure (Avior et al., 2016). Genetically identical iPSCs match somatic cell nuclear transfer–derived embryonic stem cells in their ability to form terminally differentiated cells with appropriate transcriptional, epigenetic, functional, and pharmacological features (Zhao et al., 2017b). As a result, iPSCs have become the most popular cell source for generating patient-specific somatic cells.

iPSCs can give rise to a wide variety of cell types, including cardiomyocytes (CMs) and vascular cells. iPSC-derived CMs (iPSC-CMs) provide a platform to effectively study patient- and disease-specific heart disease conditions in vitro, including Long QT syndromes (Itzhaki et al., 2011; Wang et al., 2014; Garg et al., 2018), hypertrophic cardiomyopathy (Lan et al., 2013; Seeger et al., 2019; Wu et al., 2019a), dilated cardiomyopathy (Sun et al., 2012; Wu et al., 2015; Lee et al., 2019a), Brugada Syndrome (Liang et al., 2016; Belbachir et al., 2019), left ventricular noncompaction (Kodo et al., 2016), LEOPARD syndrome (Carvajal-Vergara et al., 2010), and arrhythmogenic right ventricular dysplasia (Akdis et al., 2017; Te Riele et al., 2017). Recently, iPSCs have been used to assess whether genetic variants in individual patients are pathogenic or benign (Garg et al., 2018; Ma et al., 2018) and have proven useful in patient-specific drug screening and drug toxicity testing (Burridge et al., 2016; Matsa et al., 2016; Sharma et al., 2017, 2018). Similarly, human stem cell–derived vascular cell types such as endothelial cells (ECs) and vascular smooth muscle cells (vSMCs) are effective in modeling vascular diseases such as pulmonary arterial hypertension (PAH) (Gu et al., 2017) and for cell therapy in peripheral limb ischemia (MacAskill et al., 2018). In addition, autologous iPSC-based vaccine has arisen as a promising preventative and therapeutic strategy for combating various types of cancers, as iPSCs have shown to share with tumor cells a number of common antigens (Kooreman et al., 2018; Ouyang et al., 2019; Wang et al., 2019).

## III. Generation of Cardiac Muscle Cells from Induced Pluripotent Stem Cells

**Fig. 2.**
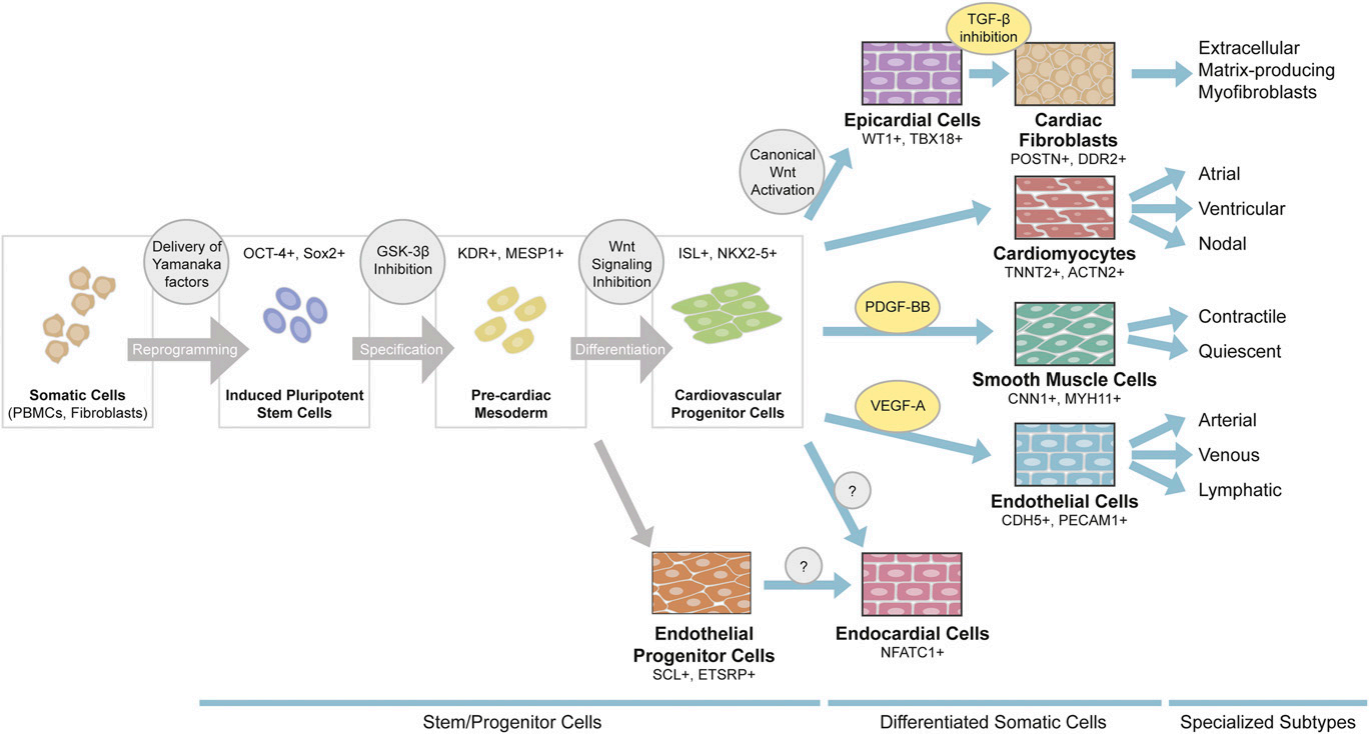
Generation of cardiovascular cells from iPSCs. Somatic cells such as dermal fibroblasts or peripheral blood mononuclear cells isolated from individual patients can be reprogrammed to iPSCs via viral delivery of the four Yamanaka transcription factors (OCT4^+^, SOX2^+^). Most commonly, iPSCs are pushed to precardiac mesodermal lineage (KDR^+^, MESP1^+^) via GSK-3*β* inhibition, and then differentiated to cardiac progenitor cells (ISL1^+^, NKX2-5^+^) by canonical Wnt signaling inhibition. Functional cardiomyocytes (TNNT2^+^, ACTN2^+^), smooth muscle cells (CNN1^+^, MYH11^+^), or endothelial cells (CDH5^+^, PECAM1^+^) are generated by treatment with unique sets of growth factors and small molecules. Epicardial cells (WT1^+^, TBX18^+^) can also be generated from cardiac progenitor cells, which can then be differentiated to cardiac fibroblasts (POSTN^+^, DDR2^+^). Generation of endocardial cells (NFATC1^+^), either from cardiac progenitors or endothelial progenitors, is still under development. Recently, methods to generate atrial, ventricular, and nodal cardiomyocytes have been described. To date it is not clear whether specialized subtypes of endothelial cells or stromal cells can be made from iPSCs.

Over the past decade, low-cost methods to derive CMs from iPSCs with high efficiency (>90%) have been developed (Lian et al., 2013; Burridge et al., 2014), enabling mass production of human cardiac muscle cells for various applications (Fig. 2). Despite such remarkable progress, a number of major challenges remain to be addressed. For example, iPSC-derived cardiovascular cells generated with current methodologies exhibit a high level of unresolved functional and transcriptomic heterogeneity, lacking the cellular maturation required for adult cell-like phenotype. Notably, the iPSC-CMs resemble fetal CMs in morphology, size, electrophysiology, and contractile and metabolic functions, necessitating the development of improved methodologies to overcome these limitations (Chen et al., 2016; Sayed et al., 2016).

### A. Induced Pluripotent Stem Cell–Derived Cardiomyocyte Differentiation

Cardiac differentiation is induced by the manipulation of specific signaling pathways essential for cardiac development and recapitulation of the embryonic cardiac differentiation process: from pluripotent stage (OCT3/4^+^, NANOG^+^), to mesodermal lineage (T^+^, MIXL1^+^), to cardiac progenitor lineage (MESP1^+^, NKX2.5^+^), and finally to relatively immature beating CMs (TNNT2^+^, MYH6^+^) (Burridge et al., 2015; Paige et al., 2015). Several key signaling pathways have been implicated in directing stepwise differentiation, including the activin A, bone morphogenetic protein (BMP)4, fibroblast growth factor 2, retinoic acid, and Wnt pathways.

In vitro cardiac differentiation protocols have dramatically improved during the past decade (Burridge et al., 2012). Initial differentiation protocols used embryoid body formation, which was inefficient and labor-intensive (Kehat et al., 2001; Mummery et al., 2012). Modern protocols use chemically defined medium and small-molecule compounds to induce CM differentiation from a monolayer of iPSCs (Lian et al., 2012; Burridge et al., 2014). First, the small-molecule glycogen synthase kinase (GSK)-3*β* inhibitor CHIR99021 is used to promote differentiation of iPSCs to mesodermal lineage, followed by a treatment with the canonical Wnt inhibitor compound IWR-1, IWR-4, XAV939, or C59 that directs differentiation of these mesodermal cells to the cardiac lineage. In these protocols, the use of serum has been eliminated and all medium components are fully defined. These differentiation methods yield robustly beating troponin T^+^ iPSC-CMs with a high efficiency at manageable cost (Burridge et al., 2014).

### B. Strategies to Generate Induced Pluripotent Stem Cell–Derived Cardiomyocyte Subtypes

Because cardiovascular diseases affect distinct chambers of the heart, it is necessary to develop methods to generate chamber-specific iPSC-CM cellular subtypes. For example, atrial CMs are required to assess the mechanisms underlying atrial fibrillation, nodal CMs for conduction disorders, and ventricular CMs for ventricular tachycardia. Recent studies have described how to modulate retinoic acid signaling during CM differentiation to derive atrial- and ventricular-like CMs (Lee et al., 2017a; Argenziano et al., 2018; Zhao et al., 2019), as well as sinoatrial nodal pacemaker cell CMs (Protze et al., 2017). These chamber-specific CMs are shown to be functionally distinct, as the calcium-handling properties of the derived atrial iPSC-CMs are different from those of ventricular or nodal iPSC-CMs (Argenziano et al., 2018). Generation of a double-reporter iPSC line that enables isolation of cells from first and second heart field can also help in distinguishing atrial and ventricular iPSC-CMs, epicardial cells, and ECs (Zhang et al., 2019b). Tissue engineering techniques enable creation of electrophysiologically distinct atrial and ventricular 3D microtissues, which have been shown to exhibit chamber-specific gene expression and drug responses to seratonin and ranolazine (Zhao et al., 2019). Furthermore, advances in single-cell sequencing have greatly aided our understanding of the iPSC-CM differentiation process and the transcriptional regulations that govern iPSC-CM subtype specification (Churko et al., 2018; Friedman et al., 2018). Lastly, efforts have been made to derive specialized populations of non-myocyte cells of the heart, such as cardiac progenitor cells (Lalit et al., 2016), epicardial cells (Bao et al., 2016; Zhao et al., 2017a), cardiac fibroblasts (Zhang et al., 2019a), and valve interstitial-like cells (Nachlas et al., 2018), from iPSCs.

### C. Challenges in Induced Pluripotent Stem Cell–Derived Cardiomyocyte Maturation

It is essential that iPSC-CMs recapitulate an adult CM phenotype for bona fide disease modeling, drug screening, and toxicity testing. However, iPSC-CMs differ from adult CMs in their morphology, contractility, metabolism, electrophysiology, and response to calcium and *β*-adrenergic stimulation. To date, researchers have attempted hormone/steroid–, culture substrate–, genetic-, three-dimensional tissue engineering–, and mechanical and electrical–based methods to mature iPSC-CMs, yet no single method has been accepted as the gold standard (Veerman et al., 2015; Bedada et al., 2016). Below we describe the major phenotypic differences between iPSC-CMs and adult CMs and discuss current methods available to address the immaturity of iPSC-CMs.

#### 1. Cellular Morphology

The iPSC-CMs generated using standard protocols display round or polygonal cellular morphology with sarcomeric disorganization, whereas mature adult CMs are rod-shaped and elongated with an aspect ratio of 5:1 to 9:1 and an anisotropic distribution of myofilaments (Gerdes et al., 1992; Ribeiro et al., 2015a). The rod-shaped morphology promotes the longitudinal alignment that mature CMs need to facilitate rapid electrical conduction and efficient muscle contraction (Kleber and Saffitz, 2014; Vreeker et al., 2014). By contrast, iPSC-CMs are highly disorganized and lack a dominant axis of myofibril alignment (Feaster et al., 2015). iPSC-CMs also exhibit a smaller surface area, as measured by membrane capacitance (Hwang et al., 2015).

Differences in organelle morphology and distributions between iPSC-CMs and adult CMs are also evident. Fetal and adult CMs have different numbers of nuclei, and ploidy per CM increases with cellular maturation (Mollova et al., 2013). iPSC-CMs are mostly mononucleated, resembling fetal CMs (Snir et al., 2003). iPSC-CMs also lack developed t-tubule networks necessary for efficient contractile function in adult CMs (Ibrahim et al., 2011).

#### 2. Contractility

iPSC-CMs differ from adult CMs in contractile and electrophysiological functions. Tulloch et al. (2011) reported that the peak twitch tension of human embryonic stem cell–derived CMs (ESC-CMs) in collagen constructs was measured at ∼0.08 mN/mm^2^, nearly 550-fold less than that of adult human myocardium. Using dynamic traction force microscopy to measure lateral force generation, Kita-Matsuo et al. (2009) reported individual cells plated on gelatin-functionalized surfaces of polyacrylamide casts contracted with an average total force of 144 nN, whereas neonatal rat ventricular myocytes (NRVMs) contracted with 222 nN total force under identical conditions. Differences in total force are attributed to the differences in generated traction, as the NRVMs displayed a higher aspect ratio (length of the long axis divided by the length of the short axis) than ESC-CMs. The ratio of axial to total force was comparable between NRVMs and ESC-CMs, indicating that variations in morphologic alignment did not contribute to polarization of force generation. More recently, cardiac tissue formed with early-stage iPSC-CMs was found to display adult-like contractility and a positive force–frequency relationship, suggesting that it may be necessary to combine three-dimensional tissue construction of iPSC-CMs with supporting cell types to achieve full contractile function (Mannhardt et al., 2016; Ronaldson-Bouchard et al., 2018).

#### 3. Sarcomeric Protein Isoforms

Isoform switching is a common developmental phenomenon in many cell types, including CMs. These switches in the expression of functional sarcomeric protein isoforms serve as useful markers in determining the maturity level of CMs. In the mammalian heart, a stoichiometric switch in the expression level of cardiac troponin I (cTnI) isoforms occurs during CM maturation. In the fetal heart, slow skeletal troponin I (ssTnI) expression predominates over cTnI expression, whereas in the adult heart cTnI expression rises and ssTnI levels decrease (Bedada et al., 2014). Similarly, ratios of myosin heavy chain (MHC) and myosin light chain (MLC) protein isoforms differ between fetal and adult CMs. In the fetal heart *β*-MHC and MLC2a expression dominates, whereas in the adult heart *α*-MHC and MLC2v expressions take over (Reiser et al., 2001). Finally, fetal CMs predominantly express the N2BA isoform of titin, a sarcomeric protein that anchors Z-discs to the M-lines of the sarcomere, whereas adult CMs primarily express the N2B isoform of titin (Opitz et al., 2004).

iPSC-CMs were shown to express ssTnI even after long-term culture, indicating the relative immaturity of the cells (Bedada et al., 2014). Ascertaining accurate ratios of *β*-MHC/*α*-MHC and MLC2a/MLC2v in iPSC-CMs is complicated due to the heterogeneity of atrial and ventricular CMs present in iPSC-CMs (Ivashchenko et al., 2013; Lundy et al., 2013). ESC-CMs were shown to express the N2BA isoform of titin, found primarily in fetal CMs (Neagoe et al., 2002; Opitz et al., 2004). Taken together, these data suggest the cTnI:ssTnI ratio and the titin isoform N2B:N2BA ratio are the preferred genetic markers to assess the maturation of iPSC-CMs.

#### 4. Mitochondrial Function and Metabolism

Mitochondria are highly abundant in the heart, comprising up to 23% of human, 28% of rat, and 32% of mouse myocardium (Schaper et al., 1985). In humans, most adult CM mitochondria are rod-shaped and aligned evenly with sarcomeres (Robertson et al., 2013). Cellular factors controlling mitochondria, including fusion factors Mfn1, Mfn2, and Opa1 and fission factor Drp1, are abundantly expressed in adult CMs (Chung et al., 2007). In comparison, ESC-CMs have thinner and fewer elongated mitochondria (Hattori et al., 2010), often clustered adjacent to the nucleus with diminished expression of mitochondrial dynamics proteins (Gherghiceanu et al., 2011).

Glycolysis is a major energy source for CMs during early cardiac development. With CM maturation and differentiation, mitochondrial oxidation, primarily in the form of fatty acid *β*-oxidation and glucose oxidation, becomes the major energy source for the adult heart. Interestingly, in certain pathologic conditions such as hypertrophic cardiomyopathy, adult cardiac metabolism switches back to a more fetal phenotype with less *β*-oxidation and more glycolysis (Lopaschuk and Jaswal, 2010). Consistent with their immature fetal-like phenotypes, ESC-CMs are predominantly glycolytic with the presence of oxidative metabolism mainly in the form of lactate oxidation (Rana et al., 2012). The transcriptome and metabolism of the ESC-CMs resemble those of the fetal CMs more than the adult CMs (Gaspar et al., 2014). Oxidative phosphorylation genes in fact are expressed in ESC-CMs at even lower levels than in fetal CMs (Ojamaa et al., 1996).

### D. Approaches to Enhance Induced Pluripotent Stem Cell–Derived Cardiomyocyte Maturation

Attempts to improve the maturation of iPSC-CMs include prolonged culture duration, treatment with tri-iodothyronine (T3) or dexamethasone (DEX), culture substrate variations, three-dimensional tissue engineering, electrical stimulation, and genetic approaches (Yang et al., 2014; Bedada et al., 2016; Tu et al., 2018). Initial efforts included prolonging the duration of iPSC-CM culture. For example, iPSC-CMs cultured for 80 to 120 days displayed greater cell size and elongation, with increased density and alignment of myofibrils (Lundy et al., 2013). Long-term cultured iPSC-CMs exhibited faster calcium-transient kinetics, enhanced contractility, and adrenergic responsiveness to isoproterenol. PKA (protein kinase A)- and proteasome-dependent signaling axis has been implicated to modulate mitochondrial respiratory chain proteins and enhance the metabolic output of long-term cultured iPSC-CMs, resulting in the increased contractility (Ebert et al., 2019). However, sarcomeric organization remains heterogeneous and does not resemble that of adult CMs, indicating a requirement of additional stimulus to achieve the full maturation.

Treatment of T3 on murine ESC-CMs was shown to upregulate NKX2-5, MLC-2v, *α*-, and *β*-MHC expression and to enhance the electrophysiological properties of the cells, increasing maximal upstroke velocity and peak amplitude of caffeine-induced calcium transients (Lee et al., 2010). Similarly, iPSC-CM differentiation in a medium containing T3 was shown to promote molecular, morphologic, and functional maturation of the cells, with a 2-fold increase in contraction stress of the cells (Ribeiro et al., 2015b). Microarray analysis showed that iPSC-CMs cultured in presence of T3 resembled second-trimester human fetal hearts, whereas control iPSC-CMs resembled first-trimester human fetal hearts (van den Berg et al., 2015). This indicates a role of T3 in the maturation of iPSC-CMs. However, it was also reported that T3 treatment did not affect the cTnI protein isoform ratio, a genetic marker for CM maturation (Bedada et al., 2014).

Synthetic glucocorticoid DEX has been shown to induce binucleation and inhibit proliferation of CMs in P4 and P7 neonatal rats by epigenetic suppression of cyclin D2 (Gay et al., 2015, 2016). Inhibition of global DNA methylation by 5-azacytidine blocked DEX-mediated downregulation of cyclin D2, suggesting that DEX acts on glucocorticoid receptors to inhibit proliferation and promote cardiac differentiation. Binucleation and suppressed proliferative activity of CMs indicate terminal differentiation and maturation of the cells.

The dynamic cellular environment of the heart tissue is not recapitulated in a two-dimensional monolayer culture of cells. To address this, several studies have used culture substrates with altered stiffness and specific patternings that more closely resemble those of the native tissue. Feaster et al. (2015) cultured single iPSC-CMs on a 0.4- to 0.8-mm–thick mattress of undiluted Matrigel for 5 to 7 days that yielded rod-shaped iPSC-CMs with increased sarcomere length when compared with traditional culture substrate of <0.1-mm–thick, diluted Matrigel. Contractility of the mattress iPSC-CMs was comparable to that of rabbit ventricular CMs and of late-stage iPSC-CMs after 80 to 120 days in culture. In addition, the mattress iPSC-CMs responded to positive and negative inotropic agents, such as myofilament Ca^+2^ sensitizer EMD57033 and Ca-channel antagonist verapamil in a concentration-dependent manner. By screening a library of polymers comprised of poly-*ε*-caprolacton (PCL), polyethylene glycol, and carboxylated PCL, Chun et al. (2015) found that culturing iPSC-CMs on 4% polyethylene glycol–96% PCL resulted in high contractility and mitochondrial function of the cells, as well as more mature gene expression patterns, including induction of MLC2v and isoform switching from ssTnI to the postnatal form cTnI.

Genetic approaches have also been promising. To mimic the switch of TnI isoforms during CM maturation of developing heart, Bedada et al. performed exogenous adenoviral-mediated gene transfer of human cTnI to iPSC-CMs, which successfully altered the cTnI:ssTnI isoform ratio (Bedada et al., 2014). Histone H3 acetylation chemically induced in iPSC-CMs by treatment with the histone deacetylase inhibitor trichostatin A increased cardiac gene expression and improved responses to potassium ion channel inhibitors. A combinatorial set of transcription factors known to induce cardiac hypertrophy (Heineke and Molkentin, 2006) such as GATA4, nuclear factor *κ*B (NF-κB), myocyte enhancer factor-2, serum response factor, and EGR1 should be considered as promising targets of genetic approaches to enhance iPSC-CM maturation.

Approaches to create three-dimensional cardiac tissue (Eder et al., 2016; Mathur et al., 2016) and to stimulate the cells with mechanical and electrical stimuli (Chan et al., 2013; Ruan et al., 2016) have also been reported. Cardiac tissue made with iPSC-CMs in fibrin hydrogel was subjected to stretch and auxotonic contractions for 4 weeks, resulting in maturation of molecular, structural, and metabolic properties of iPSC-CMs (Ronaldson-Bouchard et al., 2018). Combined stimuli promote contractility, calcium-handling protein expression, and passive mechanics of engineered cardiac tissue. Limitations of these techniques include the inability to scale up for high-throughput screening of multiple samples in parallel, which is currently being addressed via novel approaches such as the cardiopatch platform (Shadrin et al., 2017).

## IV. Generation of Vascular Cells from Human Induced Pluripotent Stem Cells

### A. Endothelial and Smooth Muscle Cell Differentiation

To generate vascular cell types such as ECs and vSMCs, iPSCs are first differentiated to mesodermal progenitors, then induced to vascular lineages by treatment with vascular endothelial growth factor (VEGF)-A to induce ECs (Patsch et al., 2015; Sayed et al., 2015; Williams and Wu, 2019) with platelet-derived growth factor (PDGF)-BB, activin A, and transforming growth factor (TGF)-*β*1 to induce vSMCs (Dash et al., 2015). Similar to vascular development in vivo, canonical Wnt activation followed by Bone Morphogenetic Protein 4 (BMP4) signaling led to formation of mesodermal progenitors most suitable for generation of ECs and vSMCs. The efficiency of pluripotent stem cells to EC differentiation is suboptimal, ranging from 10% to 35% (Patsch et al., 2015; Paik et al., 2018). Consequently, to isolate pure iPSC-EC populations, differentiated cells are subjected to magnetic-associated cell sorting with bead-conjugated CD144 antibody on the final day of differentiation. Sorted iPSC-ECs exhibit bona fide EC characteristics, including tube-like network formation, upregulation of surface adhesion molecules in response to proinflammatory cytokines, nitric oxide release, and acetylated low-density lipoprotein (LDL) uptake. Single-cell RNA sequencing of iPSC-ECs identified four subpopulations of iPSC-ECs, marked by enriched expression of CLDN5, APLNR, GJA5, and ESM1 (Paik et al., 2018). Notably, GJA5^+^ iPSC-ECs represented arterial-like cells, whereas ESM1^+^ iPSC-ECs represented cells in activated, proangiogenic state. Approaches to enrich for iPSC-ECs with arterial, venous, and lymphatic phenotype are under development (D’Souza et al., 2018; Uenishi et al., 2018). Overexpression of SIRT1 by lentiviral delivery has been reported to prolong iPSC-EC morphology and proliferative capacity by delaying early cell senescence (Jiang et al., 2015).

iPSC-vSMC differentiation relies primarily on treatment of growth factors PDGF-BB and TGF-*β*1 to yield a putatively pure populations of CD140b^+^ vSMCs without additional cell-sorting steps (Wanjare et al., 2013; Patsch et al., 2015). Different plate-coating materials and growth factor treatment have been reported to prolong culturing of iPSC-vSMCs under either synthetic or contractile conditions (Patsch et al., 2015). Cheung et al. 2012 delineated chemically defined protocols to generate origin-specific iPSC-vSMCs from intermediate lineages of neuroectoderm, lateral plate mesoderm, and paraxial mesoderm, which also resulted in highly pure populations of vSMCs with robust expression of early SMC markers ACTA2, TAGLN, and CNN1 and late SMC markers SMTN and MYH11. The resulting iPSC-vSMCs exhibited SMC functionalities such as cell migration, extracellular matrix interaction, and actin cytoskeleton organization. Generating iPSC-vSMCs in alginate hydrogel microtubes has recently been reported as a scalable method for producing contractile and less proliferative vSMCs with increased expression of ECM genes (Lin et al., 2019). The purity of iPSC-vSMCs obtained with currently available methodologies however remains questionable, given the imperfect differentiation efficiencies of other cardiovascular lineages in the absence of cell type-specific purification steps. Existing iPSC-vSMC protocols can thus benefit from additional refinements to derive the cells with higher purity, which will enable more accurate discerning of the molecular signatures of vascular diseases without the noise of unwanted cell types.

### B. Applications of Induced Pluripotent Stem Cell–Derived Vascular Cells

Patient-specific iPSC-ECs can be used to model the clinical phenotypes of vascular diseases in vitro. iPSC-ECs from idiopathic and familial PAH patients exhibited endothelial dysfunction similarly to native pulmonary arterial ECs isolated from the same patients (Sa et al., 2017). iPSC-ECs derived from familial PAH patients displayed reduced adhesion, survival, migration, and angiogenesis in comparison with healthy counterparts (Gu et al., 2017). Disease phenotypes of familial PAH patients whose BMP receptor 2 (BMPR2) mutation impaired cell adhesion and survival of ECs were recapitulated in the iPSC-ECs. Specifically, familial PAH iPSC-ECs carrying the BMPR2 mutation were not able to activate noncanonical pP38 signaling pathway in response to BMP4. CRISPR/Cas9 gene editing corrected the pathogenic BMPR2 mutation in the patient iPSC lines and rescued the pathologic phenotype. Calcific aortic valve disease patients’ iPSC-ECs were used to describe the epigenetic mechanism of NOTCH1 haploinsufficiency in activation of latent pro-osteogenic and inflammatory gene networks (Theodoris et al., 2015). iPSC-ECs can also serve as an essential component of bioengineered organoids or three-dimensional organ structures (Nakayama et al., 2015; Kim et al., 2016).

In addition, iPSC-ECs have shown promise as a source for cell therapy for ischemic disease conditions (Rufaihah et al., 2011; MacAskill et al., 2018), although poor survival and engraftment still remain major obstacles. A mixture of extracellular matrix components was used to enhance post-transplantation viability of iPSC-ECs within three-dimensional scaffolds, suggesting optimized microenvironments can improve survival and engraftment of iPSC-ECs in hypoxic conditions (Hou et al., 2016; Lee et al., 2017b). Exosomes and microRNAs secreted from iPSC-derived cardiovascular cells bear potential for cell-free therapeutic strategies (Jung et al., 2017). In addition, iPSC-ECs can serve as a toxicity-screening platform in assessing endothelial dysfunction triggered by nicotine and e-cigarette products (Lee et al., 2019b) as well as environmental toxins (Tang et al., 2017).

Similarly to iPSC-ECs, iPSC-vSMCs have been used effectively in investigating disease mechanisms of vSMC dysfunction (Maguire et al., 2017). For example, Marfan syndrome is a heritable connective tissue disorder characterized by mutations in the FBN1 gene and manifested in development of thoracic aortic aneurysm (Granata et al., 2017). iPSC-vSMCs from Marfan Syndrome patients recapitulated in vitro the pathologic presentation of the disease, such as abnormal extracellular matrix degradation and aberrant TGF-*β* signaling and apoptosis of vSMCs. Such abnormalities were rescued by gene editing of FBN1 mutation by CRISPR/Cas9, which identified the role of p38 mitogen-activated protein kinase and KLF4 in regulation of vSMC proliferation and apoptosis that leads to the pathology of Marfan syndrome (Granata et al., 2017). Likewise, iPSC-vSMCs from supravalvular aortic stenosis and Williams–Beuren syndrome patients with premature termination of ELN gene revealed that small GTPase RhoA signaling and extracellular signal-regulated kinase (ERK)1/2 activity regulate hyperproliferation of iPSC-vSMCs, responsible for pathology of the disease (Ge et al., 2012). iPSC-ECs and iPSC-vSMCs together have shown to be a critical source of three-dimensional tissue-engineered blood vessels for regenerative medicine therapeutic applications and for disease modeling of vascular diseases (Dash et al., 2016; Gui et al., 2016; Wimmer et al., 2019a).

## V. Strategies for Induced Pluripotent Stem Cell–Based Drug Discovery and Testing

### A. Methods for Cardiovascular Drug Screening

#### 1. Tissue-Specific Response

iPSCs provide the potential to rapidly screen thousands of novel drug compounds, personalize medical therapy, and repurpose medications for cardiovascular disease, as iPSC-derived cardiovascular cells can be used to screen drug libraries in a high throughput manner (Del Álamo et al., 2016; Sharma et al., 2018). For example, iPSC-CMs can test for adverse response to potential drug targets with next-generation sequencing and proteomics analysis. In a recent study, iPSC-CMs were stimulated with hydrogen peroxide to mimic ischemic injury in vitro, and short hairpin RNA–mediated gene silencing was used to validate a candidate target mitogen-activated protein kinase kinase kinase kinase-4 (MAP4K4) (Fiedler et al., 2019). Using a chemical screen, a novel small-molecule compound was found to promote survival of iPSC-CMs after ischemic injury, showing the potential in iPSC-derived cells to perform a tissue-specific high-throughput screening of drug compounds. In addition, cellular phenotyping, including calcium studies, multiple electrode arrays, and atomic force microscopy, is used to further define the patient-specific response to the candidate drug.

#### 2. Organ-on-a-Chip for Optimizing Therapy and Screening Side Effects

iPSCs can be differentiated into various tissue types and tested for side effects using biochemical and cellular phenotyping for transcriptome and proteomic profiles to predict adverse drug reactions. The use of organ-on-a-chip or organoid models will facilitate the detection of unwanted side effects, thus ensuring drugs used in clinical trials would cause minimal morbidity and mortality. iPSC-derived cardiovascular tissues can screen for effects on multiple tissue simultaneously and assess crosstalks among different cell types in response to a drug (Tzatzalos et al., 2016; Liu et al., 2018; Wnorowski et al., 2019). Taken together, the use of organ-on-a-chip models will help determine the global efficacy and safety profile for novel compounds, eliminating the need to conduct testing in primary cell culture and animal models that are considered unethical or rarely translatable clinically.

The human ether-à-go-go–related gene (hERG) encodes the inward rectifying voltage-gated potassium channel in the heart, involved in cardiac repolarization (Warmke and Ganetzky, 1994). hERG testing of pluripotent stem cell–derived CMs has the potential to identify drugs that cause QT prolongation (Curran et al., 1995; Sanguinetti et al., 1995; Sanguinetti and Tristani-Firouzi, 2006). hERG tests are an example of harnessing the power of pluripotent stem cell platforms to identify potentially fatal side effects. Although iPSC platforms have the capacity to detect cardiotoxicity, routine testing with iPSC platforms has not yet been fully integrated into the drug-screening algorithm of pharmaceutical industries, although multi-site testings are now underway (Blinova et al., 2018).

#### 3. Precision Therapies Using Induced Pluripotent Stem Cells

Meta-analysis of different drugs in the same class may also reveal that all individuals respond to drugs in the same class, a phenomenon known as a class effect. In the clinic, however, some drugs are more effective than others and have a better side effect profile. All patients are unique, and their individual responses to a drug will vary.

The cause of the variation may be due to the differences in the genetic code caused by single-nucleotide polymorphisms. The goal of precision therapy is to deliver the right medicine at the right time to the right individual. In the past, cohorts were selected based on demographic, biometrics, and cardiovascular risk factors or disease state. More recently, trials such as the Justification for the Use of Statins in Prevention: an Intervention Trial Evaluating Rosuvastatin (Ridker et al., 2008) and the Canakinumab Anti-Inflammatory Thrombosis Outcome Study (CANTOS) (Ridker et al., 2017) have used biomarkers to aid in patient recruitment. The personalized tailoring of medical therapy depends on each patient’s unique genetic makeups, lifestyle, and environmental exposures.

iPSCs offer a window into an individual’s unique response to a drug. The iPSCs retain the individual’s genetic information, and when differentiated into the appropriate tissue subtypes, can reveal unique transcriptomic, metabolomics, and proteomic profiles (Matsa et al., 2016; Lam et al., 2019). Armed with these novel biomarkers, a clinician can tailor therapy to any individual patient to maximize beneficial effects and minimize side effects.

#### 4. Predicting Responders and Nonresponders to Avoid Trial and Error

Over the past century, we have witnessed astonishing progress in the treatment of cardiovascular disease. Using animal models and primary cell cultures, mechanisms of cardiovascular disease have been uncovered and drug therapies for atherosclerosis rigorously tested in clinical trials. With the development of statins, mortality and the incidence of cardiovascular events have declined significantly. Following the mantra of lowering cholesterol levels, a multitude of clinical trials, including the Pravastatin or Atorvastatin Evaluation and Infection Therapy—Thrombolysis in Myocardial Infarction 22, a randomized trial of cholesterol lowering in 4444 patients with coronary heart disease, the Scandinavian Simvastatin Survival Study (4S), and Myocardial Ischemia Reduction with Aggressive Cholesterol Lowering, have demonstrated the tremendous power of statins to alter the trajectory of atherosclerotic disease (Schwartz et al., 2001; Cannon et al., 2004; Pedersen et al., 2004). The current standard of care for primary prevention is to place a patient who has moderate or high risk on a statin, and all patients should be on a statin for secondary prevention (Stone et al., 2014). In the clinic, however, we find patients who do not respond as well to statin therapy, and many patients have reported adverse side effects.

Although statin therapy is essential for preventing cardiovascular deaths and improving survival, the implementation is difficult and often frustrating for both patients and clinicians. Similarly, patients with atrial fibrillation are prescribed rhythm or rate control strategies based on patients’ symptoms and preferences. Using an iterative process, clinicians and patients work together to find a drug with the best risk-to-benefit ratio. More broadly, iPSCs could identify subpopulations that would respond to therapy and experience the benefits of a drug while minimizing the adverse effects. Using iPSC-derived skeletal myoblasts, high-throughput assay for screening statin-induced myopathy was developed to test for mitochondrial integrity and cytoplasmic integrity (Klaren and Rusyn, 2018). With lowered cost of the assay and increased accessibility of iPSC-derived cells, such platform will be critical in distinguishing responders versus nonresponders in vitro.

#### 5. Optimizing Clinical Trials

Patients who meet clinical trial entrance criteria should respond appropriately to therapy. The dilemma is that many trials, despite being adequately powered, fail to show improvement or even demonstrate adverse events in the enrolled patients. Clinical trials are designed with entrance and exclusion criteria to select responders to treatment and minimize adverse side effects. For example, the Cardiac Arrhythmia Suppression Trial (CAST) was designed to show the efficacy of antiarrhythmic drugs in suppressing ventricular arrhythmias in ischemic cardiomyopathy (Echt et al., 1991). However, part of the outcome was an unexpected increase in death due to arrhythmic death and shock. Because of their unique advantages, iPSCs could be used to predict hazard and further enhance exclusion criteria to avoid adverse effects (Kopljar et al., 2018; Wu et al., 2019b).

There already exist several examples of iPSC-CMs in modeling drug-induced toxicity with the potential to guide clinical management. Tyrosine kinase inhibitors (TKIs) are a powerful family of cancer therapies, but, unfortunately, some patients exhibit susceptibility to cardiotoxicity and arrhythmias from TKIs. Sharma et al. (2017, 2018) eloquently used human iPSC-CMs to screen in vitro for specific TKIs that cause cardiotoxicity and discovered that VEGF/PDGF receptor inhibition was important for causing cardiotoxicity. Pharmaceutical companies can likewise benefit from preclinical screening approaches with iPSC-CMs to identify compounds that cause cardiotoxicity and morbidity and to reduce time and funding required for clinical trials (Strauss and Blinova, 2017; Fermini et al., 2018).

The cardiotoxic effects of a drug may be patient-specific due to inherited factors. In another study, Burridge et al. (2016) used patient-specific iPSC-CMs to predict whether an individual would develop cardiotoxicity to anthracyclines. As such, the use of iPSC-CMs can guide patient-specific treatment and identify drugs are effective in some individuals and harmful in others, with the potential to rescue drugs that were previously discarded as unsafe due to malignant side effects in a specific cohort of individuals. Greater detail on cancer therapy–induced cardiotoxicity is discussed in *Section VI* of this review.

The cost of developing a drug is approximately $ 2.6 billion, with over 80% of the funds spent in the clinical trials phase of drug development and with approximately seven of eight drugs failing to make it into the clinic (DiMasi et al., 2016). iPSC-based drug screening boasts the power to identify drug toxicities, such as anti-arrhythmics that paradoxically have caused more cardiovascular death. By quickly failing at the preclinical stage, a pharmaceutical company can avert a clinical trial and save billions of dollars that can be repurposed.

Traditional biomarkers require the use of biometrics, serum proteins, and imaging. In this omics era of today, future trial designs may incorporate individual profiles from genetics, transcriptomics, metabolomics, and proteomics. Using this highly tailored approach, a drug will be more efficacious in receptive populations. Clinical trials that may have shown a signal for a positive response using traditional selection criteria would be more likely to be clinically significant.

### B. Strategies for Induced Pluripotent Stem Cell–Based Drug Discovery

**Fig. 3.**
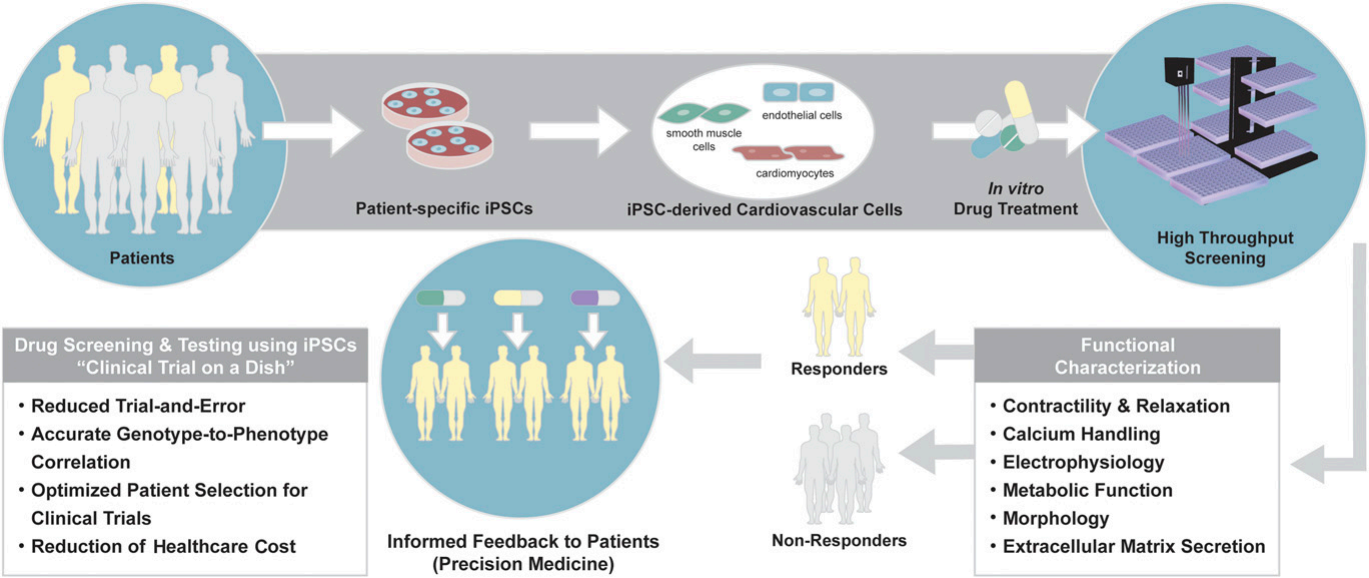
Drug screening and testing using patient-specific iPSCs. Patient-specific iPSCs provide a platform for personalized drug screening and testing. iPSCs generated from individual patients are differentiated into cardiovascular cells of interest and subjected to drug treatment in vitro. High-throughput methods allow functional characterization of the cells during or after drug treatment, from which responders vs. nonresponders and the side effects of the drug are identified. This information allows informed feedback to the patients regarding the drug’s efficacy and toxicity based on the individual responses to the drug. As such, patient-specific iPSC-based drug screening and testing provide accurate genotype-to-phenotype correlation that results in precise drug discovery and reduces the stressful and costly trial-and-error process for the patients, not to mention significantly reducing the cost of health care.

Drug screening and testing using patient-specific iPSCs enable the realization of “clinical trial in a dish” to reduce the exorbitant cost and inefficiency of the current drug development pipeline in the United States and other countries. The use of disease- and patient-specific iPSCs in drug screening and testing will also reduce trial and error and optimize patient selection for clinical trials, by accurately predicting responders versus nonresponders with greater accuracy (Fig. 3).

#### 1. In Silico Prediction for Candidate Drugs

In silico analysis predicts compounds binding to drug target receptors. The structure of a receptor must be known to allow a known ligand to facilitate the modeling of binding with the use of a compound library. Designed algorithms can predict binding to putative ligands, which must be subsequently validated in vitro or in vivo (Lionta et al., 2014; Kernik et al., 2019). Use of iPSCs can facilitate in silico drug discovery by providing a platform to validate novel drugs in a high throughput manner. Moreover, iPSC disease modeling may uncover novel targets. Thus, iPSCs not only can validate drugs but also find new targets for in silico drug discovery.

#### 2. Repurposing Drugs for Cardiovascular Disease

An existing drug may be repurposed for a new indication. For example, colchicine is an anti-inflammatory drug for gout, which found off-label use in other inflammatory conditions such as pericarditis and postoperative pericarditis syndrome (Imazio et al., 2005, 2013). Existing drugs can be repurposed for other clinical indications, with the use of iPSCs to facilitate screening existing FDA-approved drugs for cardiovascular indications, helping to reduce or eliminate the costly preclinical screening normally needed to ensure safety and efficacy.

#### 3. Screening Novel Compounds

Drug screening can involve creating new compounds from scratch or using existing molecules to design novel compounds with improved efficacy or side effect profile. Rational drug design can be used to synthesize a multitude of compound (Drews, 2000). The efficacy and side effect profile of each compound, however, must be validated in a biologic model before expensive and lengthy clinical trials. Dronedarone is an example of premodifying a drug structure in an attempt to minimize toxicity and to retain its pharmacological benefit. The result, however, was a less potent antiarrhythmic drug with more adverse effects than the original (Connolly et al., 2011). The use of iPSCs therefore can allow screening of candidate drugs for toxicity and adverse effects before the initiation of clinical trials to save time and cost.

### C. Challenges of Implementing Pluripotent Stem Cell Platforms in Industry

Despite the many advantages for drug discovery, pluripotent stem cells have not yet gained traction in the pharmaceutical industry. ESC-based strategies face a significant stigma with ethical concerns. The use of iPSCs can avoid this issue and has many advantages over ESCs. iPSCs retain the genetic information of an individual and provide a unique opportunity to advance personalized medicine. In regenerative therapy, the use of iPSCs may eliminate the need for immunosuppression, which is needed for the case of ESCs. Genome editing can further augment the power of iPSCs to correct mutations causative of the disease. Although gene therapy is not yet feasible, gene editing will uncover novel mechanisms of disease and targets for small-molecule therapies, which when combined with iPSCs will advance the development of novel personal therapies.

However, iPSC-based platforms are not yet high throughput, remain expensive, and require storage for the delivery of regenerative therapies in a timely manner. The cost of drug discovery with iPSC platforms could be enormous, as each person would require a blood sample or tissue sample to be reprogrammed and subsequently differentiated. This individualized approach inevitably takes time because the reprogramming and differentiating process can take up to 6 months for each individual, not counting the time needed for recruiting patients into the study. Making the drug discovery process high throughput may thus prove more difficult than expected, as disease modeling likely will add considerable complexity to the drug-screening process, and titrating for each individual may require multiple iterations.

Solomon et al. (2015) and Lee et al. (2018) have devised a strategy to partially circumvent the challenges of drug screening and regenerative therapy with iPSCs by generating banks of cell lines, in which a diverse set of iPSC lines from healthy individuals with varying combinations of commonly present HLA alleles is banked. The iPSCs with common but not identical HLA profile will be differentiated and may be used for regenerative therapeutic solution. The resulting iPSC-derived cells will nonetheless require immunosuppression, but there is a potential for individualized treatment free of ethical concerns.

## VI. Cancer Therapy–Induced Cardiotoxicity Screening

**Fig. 4.**
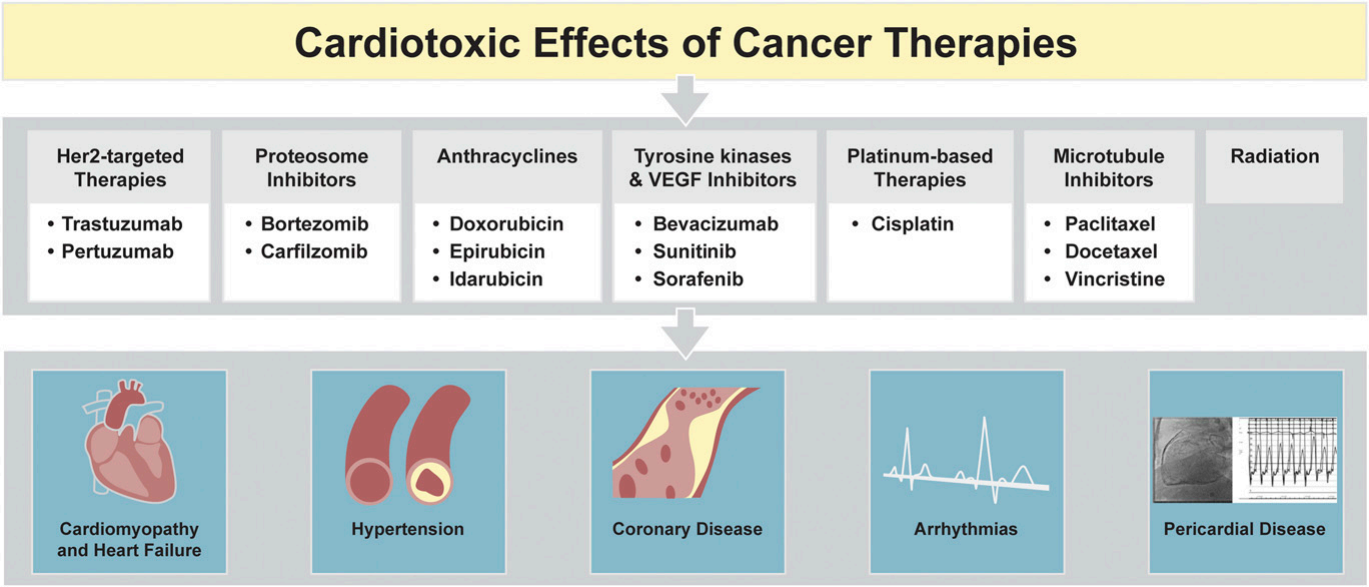
Cardiotoxic effects of cancer therapies. Although a number of targeted and untargeted cancer therapies have shown efficacy in cancer treatment, these therapies also cause cardiotoxic effects that can result in profound cardiovascular complications. Chemotherapies, including anthracyclines such as doxorubicin, tyrosine kinase inhibitors, Her2-targeted therapies, and proteosome inhibitors, are associated with vascular dysfunction and CM damage that can lead to heart failure. Platinum-based therapies, microtubule inhibitors, and radiation therapy have also been linked to arrhythmias and pericardial disease. The pathobiology or the disease mechanisms of such cardiotoxic effects of various cancer therapies have not been elucidated and must be addressed in a patient-specific manner.

From 1953 to 2013, 14% of the drugs were withdrawn from market due to serious cardiovascular adverse events (Onakpoya et al., 2016). These include a number of cancer drugs and therapies reported to cause adverse cardiovascular side effects, leading to CM damage and heart failure, arrhythmias, hypertension, and coronary artery disease (Lenneman and Sawyer, 2016) (Fig. 4). The current approach to testing pre-market compounds is not effective in gauging patient-specific responses (Magdy et al., 2018). The use of patient-specific iPSCs in drug safety testing and screening will thus be critical in advancing these approaches (Sayed et al., 2019).

### A. Doxorubicin-Induced Cardiotoxicity

The anthracycline antibiotic doxorubicin is one of the oldest, yet most effective anticancer agents. Doxorubicin is used to treat a wide range of malignancies, including 50% to 60% of breast cancer and 70% of childhood cancer treatment protocols. However, since the use of doxorubicin began, the presence of a dose-dependent complication of cardiotoxicity has been recognized (Vejpongsa and Yeh, 2014; Yeh and Chang, 2016). Doxorubicin-induced cardiotoxicity (DIC) is classed as acute, developing immediately during treatment; chronic, developing within the 1st year; or late-onset, occurring up to 10 years after treatment (Zamorano et al., 2016). Cardiotoxicity can range from asymptomatic reductions in left ventricular ejection fraction to symptomatic heart failure (New York Heart Association class III to IV). These patients often make poor heart transplant candidates because of their history of cancer (Shakir and Rasul, 2009). The dose-dependent cardiotoxicity of doxorubicin is well established (Lefrak et al., 1973; Von Hoff et al., 1979). Initial reports documented the highest risk for DIC at doses above ∼450 mg/m^2^. With improved methods of detecting subtle changes in cardiac function, the incidence of DIC is now thought to be much higher, occurring in up to 65% of long-term survivors of breast cancer, even at doses as low as 228 mg/m^2^ (Kremer et al., 2002; Swain et al., 2003). A study of three anthracycline trials found subclinical cardiotoxicity in patients rather than true latency between drug exposure and onset of symptoms (Grenier and Lipshultz, 1998; Swain et al., 2003). Furthermore, several studies have now shown that as many as 16% of children with these abnormalities will develop subsequent clinical heart failure with a mortality rate as high as 72% (Lefrak et al., 1973; Felker et al., 2000; Lipshultz et al., 2008).

Despite more than 50 years of research, the genetic and molecular mechanisms of DIC remain unclear (Vejpongsa and Yeh, 2014; Abe et al., 2016; Yeh and Chang, 2016). To date, three major inter-related mechanisms for cardiotoxic effects of doxorubicin have been proposed as follows: 1) generation of reactive oxygen species and subsequent membrane damage; 2) inhibition of topoisomerase II-*β* (TOP2B) and topoisomerase I mitochondrial; and 3) modulation of intracellular calcium release. iPSC-CMs from DIC patients have been used to recapitulate the phenotype and elucidate the molecular mechanisms of the drug’s adverse effects (Magdy et al., 2018). iPSC-CMs from healthy controls, breast cancer patients who received doxorubicin treatment without cardiotoxic side effects, and breast cancer patients with DIC symptoms were generated, and their transcriptomic profiles and functional differences were compared (Burridge et al., 2016). Upon doxorubicin treatment, iPSC-CMs of doxorubicin-sensitive patients displayed sarcomeric disarray, arrhythmic beating, and increased rate of apoptosis in comparison with control iPSC-CMs. Doxorubicin-sensitive iPSC-CMs also showed significantly increased reactive oxygen species levels after 24 hours of doxorubicin treatment, as indicated by increased levels of whole-cell hydrogen peroxide and antioxidant glutathione, a cellular oxidative stress response marker. RNA-seq of iPSC-CMs from the three patient groups showed a significant increase in expression levels of program cell death and p53 downstream pathway genes.

**Fig. 5.**
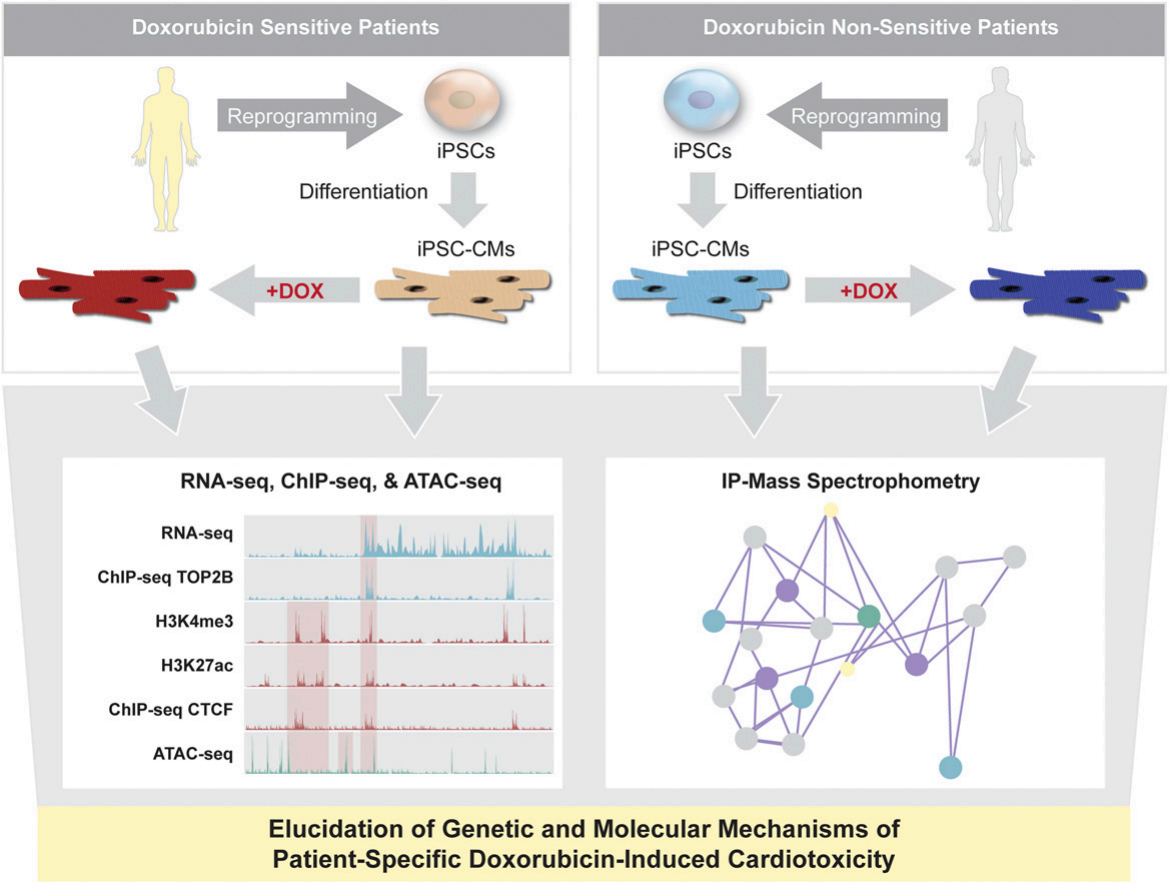
Elucidating mechanisms of doxorubicin-induced cardiotoxicity. Doxorubicin has shown cardiotoxicity for some patients. The patient-specific disease mechanisms of doxorubicin-induced cardiotoxicity remain poorly understood, and the direct and indirect effects of doxorubicin on CMs have not been clarified. Previous studies demonstrated that susceptibility of TOP2B to its target genes is dynamically modulated by doxorubicin. Using iPSC-derived CMs from doxorubicin-sensitive and nonsensitive patients, the transcriptional machinery of TOP2B can be determined by combining chromatin immunoprecipitation-seq, RNA-seq, transposase-accessible chromatin-seq, and immunoprecipitation–mass spectrometry. Cross-analysis of RNA-seq and chromatin immunoprecipitation-seq will further allow identification of direct target genes of TOP2B, which can be confirmed by chromatin immunoprecipitation-seq of TOP2B in human heart tissue. Verification of cardiac-specific target genes of TOP2B will lead to effective patient-specific therapeutics to combat doxorubicin-induced cardiotoxicity.

To develop effective therapeutics to treat DIC, it is necessary to fully elucidate the underlying genetic and molecular mechanisms of the drug’s side effects on CMs. Previous studies demonstrated that the susceptibility of TOP2B to select its target genes is dynamically modulated by treatment of doxorubicin (Zhang et al., 2012). These results led to a hypothesis that TOP2B is a key regulator of DIC and is differentially regulated in doxorubicin-sensitive and nonsensitive patients. However, the underlying mechanisms by which TOP2B distributes to chromatin and transcriptionally regulates specific target genes are still unidentified. Using the iPSC-CMs generated from the doxorubicin-sensitive and nonsensitive patients, a combination of techniques such as RNA-seq, chromatin immunoprecipitation-seq, transposase-accessible chromatin-seq, and immunoprecipitation–mass spectrometry can be employed to determine the role of TOP2B in transcriptional regulation of its target genes, such as *CTCF*, *RAD21*, and *DINO* (Uusküla-Reimand et al., 2016) in DIC patient-specific iPSC-CMs (Fig. 5).

### B. Tyrosine Kinase Inhibitor–Induced Cardiotoxicity

The development of small-molecule TKIs has led to a dramatic increase in life expectancy for cancer patients suffering from leukemias, carcinomas, and melanomas. TKIs inhibit the kinase phosphorylation activity of hyperactive receptor tyrosine kinases, thereby halting the enhanced cell survival, proliferation, and migration phenotypes that are hallmarks of cancer progression. However, several TKIs are linked to severe cardiovascular side effects (Moslehi and Deininger, 2015; Herrmann, 2016). Similar to DIC, the mechanism of TKI-driven cardiotoxicity remains poorly understood.

Currently, more than 80 TKIs are in development, with cancer being the major clinical indication. The first TKI approved was imatinib, followed by erlotinib, sorafenib, sunitinib, dasatinib, lapatinib, nilotinib, gefitinib, pazopanib, axitinib, and bosutinib. Sunitinib, sorafenib, and ponatinib target the VEGF receptor and have been noted to induce hypertension, arterial occlusions, and venous thromboembolisms (Eskens and Verweij, 2006) by adversely affecting ECs (Chu et al., 2007) and pericytes (Chintalgattu et al., 2013). Whereas preclinical screening with animal models is not able to accurately identify the TKIs that cause cytotoxicity, the iPSC technology overcomes this limitation by providing patient-specific CMs and vascular cells *en mass*.

Notably, a high-throughput screening of 21 TKIs on iPSC-CMs showed that nilotinib and crizotinib led to the highest level of cytotoxicity, whereas sunitinib and erlotinib generated the least degree of adverse cellular effects to CMs (Sharma et al., 2017). CM impedance assays have similarly been used to screen for TKIs with high cardiotoxic potential (Peters et al., 2015). Lamore et al. (2017) reported 30 of 65 TKIs investigated affected CM contraction by regulating expression of genes related to calcium flux and action potential duration. By taking into account various functional parameters of iPSC-CMs obtained from the high-throughput screening, a cardiac safety index can be generated and used as a predictive quantitative metric for patient-specific cardiotoxicity of TKIs (Sharma et al., 2018). Using iPSC-derived vascular cells, the exact effects and patient-specific mechanisms of TKIs on hypertension and vasculopathies should be investigated in future studies.

### C. Human Epidermal Growth Factor Receptor Inhibitor–Induced Cardiotoxicity

Human epidermal growth factor receptor–targeted therapies such as trastuzumab, pertuzumab, and lapatinib have shown a great promise in management of breast cancer and are generally well-tolerated (Florido et al., 2017). Trastuzumab therapy has nonetheless been reported to elicit cardiotoxicity by decrease in left ventricular ejection fraction with or without clinical diagnosis of heart failure, which manifests during the course of treatment with the potential of reversibility in cardiac dysfunction when withdrawn (Ewer et al., 2005). In vitro treatment of clinically relevant doses of trastuzumab to iPSC-CMs caused significant impairment in contractility and calcium handling of the iPSC-CMs, but not cytotoxic effects or disorganization of the sarcomeres reported to be triggered by anthracycline treatment (Kitani et al., 2019). The study has furthermore elucidated that trastuzumab led to mitochondrial dysfunction by altering cardiac energy metabolic pathways. Interestingly, iPSC-CMs generated from breast cancer patients with trastuzumab-induced cardiotoxicity were shown to be more vulnerable to trastuzumab therapy with decreased contractility and impaired oxygen consumption and autophagy activity, underlying a possibility of genetic predisposition for trastuzumab-induced cardiotoxicity. Recent findings have ben shown metformin, an anti-diabetic medication, can render cardioprotective effects by activating AMP-activated protein kinase (AMPK) signaling (Gundewar et al., 2009; Yin et al., 2011), suggesting a potential repurposing of metformin as a prevention or treatment of trastuzumab-induced cardiac dysfunction in patients with cancer. As such, patient-specific iPSC-CMs can provide important clues to cellular and molecular differences in cardiotoxicity induced by different classes of chemotherapeutic agents. Future studies using iPSC platform are necessary to elucidate the potential cardioprotection by concurrent treatment of conventional heart failure medications with trastuzumab, such as *β*-blockers or renin-angiotensin inhibitors (Bosch et al., 2013; Pituskin et al., 2017).

## VII. Therapeutics for Cardiac Disease

### A. Arrhythmia

Dronedarone, the de-iodinated derivative of amiodarone, was touted as having all the beneficial effect of amiodarone without the associated toxicities (Connolly et al., 2011). The Dronedarone Versus Amiodarone for the Maintenance of Sinus Rhythm in Patients with Atrial Fibrillation trial (Le Heuzey et al., 2010), however, found that dronedarone was not as efficacious as amiodarone at keeping patients in sinus rhythm. For maintenance of sinus rhythm, dronedarone was found to be equivalent to sotalol or flecanide. Promising post hoc analysis from ATHENA (Hohnloser et al., 2009) suggested that dronedarone could prevent cerebrovascular accidents, but the composite of cerebrovascular accidents, acute coronary syndrome, and cardiovascular death was conclusively refuted in the ANDROMEDA and PALLAS trials (Connolly et al., 2011), which clearly showed that dronedarone was associated with more thrombotic events and adverse events in heart failure and chronic atrial fibrillation (Køber et al., 2008). Dronedarone thus failed to live up to its billing as a rhythm control medication equally efficacious to amiodarone (Touboul et al., 2003; Singh et al., 2007; Davy et al., 2008); moreover, dronedarone was found to be deleterious by resulting in heart failure and chronic atrial fibrillation and was in no way superior to anticoagulation for preventing thromboembolic events (Køber et al., 2008; Le Heuzey et al., 2010; Connolly et al., 2011).

Drugs like dronedarone cost billions of dollars to develop and test in expensive clinical trials. Preclinical testing with iPSC-CMs and cardiac organoids could have averted the resulting financial catastrophe. Combined with multiple electrode array, atrial-specific iPSC-CMs and engineered heart tissues can now be used to tailor drug therapy for individual patients (Laksman et al., 2017; Lee et al., 2017a; Lemme et al., 2018). Avoiding the iterative process of finding optimal medical therapy for atrial fibrillation with reduced cost to society will help more patients earlier and reduce their morbidity and mortality. Indeed, iPSC-based technology represents a significant improvement in our ability to tailor drug therapy in a notoriously difficult to manage population that accounts for approximately $10,100 to $14,200 in the United States from data compiled from 1990 to 2010 (Wolowacz et al., 2011).

Moreover, using the iPSC-based platforms, new targets for drug therapy can be discovered for atrial fibrillation (Devalla et al., 2015; Cyganek et al., 2018). Unlike animal models that exhibit vastly different heart rates from humans, iPSC-based cardiovascular platforms reflect the human physiologic heart rate, increasing the likelihood of translational success of compounds under testing.

### B. Cardiomyopathy

Cardiomyopathy is defined as a weakness of the heart muscle. Cardiomyopathy patients suffer from dyspnea, edema, and renal failure and succumb to pump failure or sudden cardiac death if medical and device therapy is not implemented with disease progression (Braunwald, 2017). In the absence of ischemic or valvular causes, cardiomyopathies are broadly categorized into dilated, restrictive, hypertrophic, or arrhythmogenic right ventricular cardiomyopathy. The mechanisms of nonischemic cardiomyopathies are largely unknown, although recent studies suggest that single-nucleotide polymorphisms may contribute to the development of these cardiomyopathies (Hershberger et al., 2018). Despite reductions in symptoms, hospital admissions, and mortality, medical therapy for cardiomyopathy has suffered from a dearth of new drug development.

Dilated cardiomyopathy (DCM) has been attributed to mutations in sarcomere and sarcolemma-related genes, including titin, lamin A/C (LMNA), MHC, and cardiac troponins (Buikema and Wu, 2017). For instance, Lee et al. (2019a) showed iPSC-CMs from DCM patients with mutations in LMNA gene displayed calcium-handling dysfunction, leading to arrhythmic phenotype at the single-cell level. The study further reported aberrant activation of PDGF-signaling pathway in mutant iPSC-CMs to be responsible for the pathologic phenotype, which is rescued by pharmacological and molecular inhibition of PDGF receptor B. In iPSCs derived from an independent proband of LMNA-associated DCM, Siu et al. (2012) found pharmacological inhibition of ERK1/2 pathway with mitogen-activated protein kinase kinase 1/2 inhibitors to attenuate proapoptotic phenotype of DCM iPSC-CMs.

Hypertrophic cardiomyopathy (HCM) is an abnormal thickening of the ventricle that causes microinfarction, outflow tract obstruction, and scarring, resulting in arrhythmias (Marian and Braunwald, 2017). Patients have symptoms of chest pain, dyspnea, and syncope, and sometimes sudden cardiac death. A recent study found that a premature stop codon in MYBPC3 leads to HCM via chronic activation of nonsense-mediated decay (Seeger et al., 2019). Using CRISPR technology, isogenic mutation-corrected iPSC lines were generated to serve as a control to HCM patient-derived iPSCs. Comparison of the mutant and isogenic control lines showed that HCM iPSC-CMs display abnormal calcium-handling properties without haploinsufficiency of MYBPC3, suggesting that early pathophysiological processes at the molecular level may precede the disease development. Recently, Wu et al., 2019a reported novel methods to measure impaired diastolic dysfunction by a combination of calcium imaging and traction force microscopy in various familial HCM lines that carry mutations in MYH7, MYBPC3, and TNNT2 genes. Simultaneous recording of calcium transient and contractile stress by functional imaging showed elevated diastolic intracellular calcium levels and enhanced myofilament calcium sensitivity in HCM iPSC-CMs in comparison with those from healthy donors or DCM patients. The study furthermore showed calcium channel blockers such as verapamil and dilitiazem and late sodium channel blockers such as ranolazine and electlazine can reequilibrate calcium homeostasis and partially restore diastolic function in HCM iPSC-CMs. In another study, bulk RNA sequencing of iPSC-CMs from healthy donors and HCM patients after chronic exposure of four individual calcium channel blockers (nifedipine, amlodipine, dilitiazem, and verapamil) elucidated the patient-specific and drug-specific transcriptomic signatures, suggesting that the individual response may be related to ability to compensate for the inhibition on calcium entry (Lam et al., 2019). In addition, Jaffré et al. (2019) showed aberrant ERK5 and mitogen-activated protein kinase kinase 1/2 signaling pathways to be responsible for RAF1-associated Noonan syndrome–induced HCM. As such, the use of CRISPR gene editing and multimodal sequencing techniques in iPSCs has become a powerful tool in directly evaluating the pathogenic role of a mutation or a variant in cardiomyopathies (Musunuru et al., 2018).

Development of therapies for left ventricular noncompaction (LVNC), a condition increasingly recognized as a cause of cardiomyopathy especially in children, is also in dire need. Characterization of iPSC-CMs from LVNC patients carrying a mutation in cardiac transcription factor TBX20 has shown that abnormal activation of TGF-*β* signaling leads to defects in proliferation of CMs (Kodo et al., 2016). Another investigation of familial LVNC patients underlined the contribution of NKX2-5 variant as a genetic modifier of LVNC in conjunction of missense mutations in MYH7 and MKL2 genes, confirmed by genome-edited mouse models and patient-derived iPSC-CMs (Gifford et al., 2019). The data presented in these studies suggest a need for development of therapeutics that simultaneously target all pathologic variants to effectively treat complex genetic diseases such as LVNC. Generation of cardiac-specific fibroblasts from human iPSCs will also be critical in assessing patient-specific pathophysiology of cardiac fibrosis that occurs in tissue-ablating conditions, such as ischemic cardiomyopathy (Zhang et al., 2019a). As such, with improved understanding of the mechanisms of cardiomyopathies and advancement in high-throughput screening techniques, patient-specific iPSCs will continue to prove valuable in screening for novel therapeutics that reverse the disease phenotype and ameliorate cardiac function.

### C. Regenerative Medicine

The use of iPSCs for cardiovascular regenerative medicine provides a limitless source of patient-specific cells that can repair and/or even replace damaged organs (Abou-Saleh et al., 2018; Chien et al., 2019). iPSCs are considered autologous and believed to not require immunosuppression, and gene editing can reverse disease phenotype by correction of the pathogenic mutation or the variant. Nonetheless, translating iPSC regenerative therapy from bench to clinical practice faces many hurdles, one of which is that the mass generation of iPSC-derived cells requires significant time and cost. iPSC-derived cells also exhibit batch variability and lack cellular maturity.

Consequently, FDA approval would likely require that iPSC-derived cell therapy fully address the issues of arrhythmogenicity, tumorigenicity, and immunogenicity (Neofytou et al., 2015). Continued advances in biomaterial and tissue engineering may enable iPSC-derived cardiovascular tissue to better circumvent these challenges in regenerative medicine applications (Huang et al., 2018).

## VIII. Therapeutics for Vascular Disease

### A. Atherosclerosis

In both primary and secondary prevention studies, statins are the cornerstone of medical therapy. By lowering cholesterol, statins have been effective in reducing cardiovascular events and improving survival. Numerous clinical trials in secondary prevention have described the dose-dependent reductions in LDL levels, correlated with improved survival (Ridker et al., 2008). The emergence of proprotein convertase subtilisin/kexin type 9 (PCSK9) inhibitors has made it possible to further lower LDL (Sabatine et al., 2017).

Although statin and PCSK9 therapies strive to lower cholesterol levels, inflammation also plays a significant role in the pathophysiology of cardiovascular disease. The CANTOS trial revealed that the anti–interleukin-1*β* antibody Canakinumab could further reduce cardiovascular death and mortality beyond optimal treatment with existing medical management that includes the use of statins and PCSK9 inhibitor (Ridker et al., 2017).

iPSC-based drug screens may identify novel drugs that can target cholesterol biosynthesis. For example, the iPSC-derived hepatocyte-like cells have been used to study cardiac glycosides as an approach to reduce hepatocyte production of apolipoprotein B and consequently to treat hypercholesterolemia (Cayo et al., 2017). Moreover, iPSC-ECs and iPSC-vSMCs can be used to model atherosclerosis and test the efficacy of drugs that target the inflammatory changes associated with endothelial dysfunction and plaque formation.

### B. Hypertension

Stage I hypertension is defined by systolic blood pressure >130 mm Hg or a diastolic pressure >80 mm Hg, and stage II hypertension with a systolic pressure >140 mm Hg or a diastolic >90 mm Hg (Whelton et al., 2018). The Antihypertensive and Lipid-Lowering Treatment to Prevent Heart Attack Trial study demonstrated the importance of lowering blood pressure and reducing cardiovascular risk by using appropriate medications (ALLHAT Officers and Coordinators for the ALLHAT Collaborative Research Group: The Antihypertensive and Lipid-Lowering Treatment to Prevent Heart Attack Trial, 2002). The study did allow the caveat that underlying comorbidities may direct drug therapy, such as using an adrenocortical extract inhibitor in the context of diabetes and concomitant hypertensive disease. Patients often require more than one hypertensive agent to control their blood pressure (Jamerson et al., 2008). Patient-specific differences can explain why individuals respond to medications differently.

Hypertension is a disease primarily of the vascular wall, and therefore iPSC-derived vascular cells may be helpful in identifying new targets and developing new drug therapies for this condition (Biel et al., 2015). Moreover, iPSC platforms could be used to test these compounds and to tailor therapy to the individual to avoid the costly and inefficient iterative process of trial and error otherwise required for finding the optimal antihypertensive regimen.

### C. Aortopathy

Aortopathy is a progressive disease of the vessel wall that results in cystic medial necrosis and aneurysm or rupture of the vessel over time (Liddicoat et al., 1975). The aorta is normally an elastic structure, but defects in collagen synthesis and connective tissue can make the vessel more susceptible to shear stress from blood ejected from the heart. Traditionally, aortopathies are managed with *β*-blockers that reduce shear stress on the aorta. Studies using mouse models had suggested that inhibitors of TGF-*β* signaling could help delay the progression of Marfan syndrome (Neptune et al., 2003). Marfan syndrome patient-derived iPSC-vSMCs recapitulated the clinical phenotype in vitro and confirmed the critical role of TGF-*β* signaling in regulation of proliferation and apoptosis of aortic vSMCs (Granata et al., 2017). An independent study reported transcriptomic analysis that showed Marfan syndrome patient-derived iPSC-vSMCs to be more synthetic and less contractile than the wild-type counterpart (Park et al., 2017). Angiotensin receptor blockers were found to be superior in mice (Habashi et al., 2006). However, the overall outcome of the clinical trial was negative, highlighting the overarching problem with mouse models that do not translate into viable clinical therapies (Lacro et al., 2014).

The progression of aortopathy to dilatation, aneurysm, and possible rupture makes regenerative therapy a welcome option to surgery, either planned or emergent, which is associated with significant morbidity and mortality. iPSC-vSMCs can thus be used to discover new biomarkers of aortopathy, which are expensive and difficult to detect without serial imaging. Using an iPSC-vSMC array, it may be possible to develop new small molecular inhibitors of central medial necrosis that is pathogenomic for aortopathy. Furthermore, to best reflect the physiologic function and region-specific blood flow hemodynamics in aortopathic conditions, it is necessary to develop a vascular surrogate system that cocultures iPSC-derived vascular, stromal, and immune cell types under atheroprone hemodynamics. Such efforts are currently under development to model changes in hemodynamics and response to vascular inflammation or drug treatments using iPSC-derived vascular cells (Collado et al., 2017).

## IX. Therapeutics for Cardiometabolic Disease

**Fig. 6.**
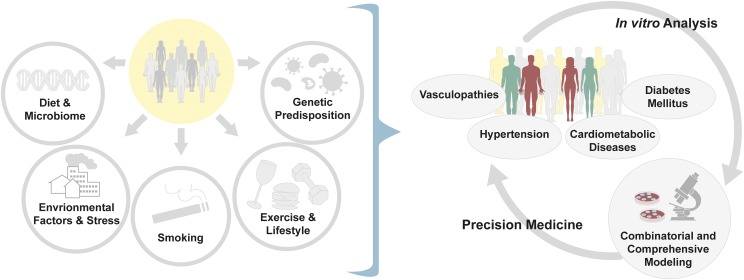
Multifaceted etiology of complex cardiovascular diseases. Cardiovascular diseases, such as hypertension and vasculopathies, and cardiometabolic diseases from diabetic conditions are complex in nature, with highly multifaceted disease etiology. The known causal factors of disease include, but are not limited to, the following: genetic predisposition, exposure to environmental pollutants, stress, diet and microbiome changes, smoking, and daily routines such as aerobic activities. To best model such complex diseases, a combined analysis of transcriptome, metabolome, epigenome, and function of patient-specific iPSC-derived cells challenged with the factors in question must be performed. The results of these meta-analyses will provide precise characterization of the patient’s disease phenotype that optimizes the design of patient-specific therapeutics.

Cardiometabolic diseases and vasculopathies stem from a combination of factors, including environmental exposure, stress, smoking, poor diet, and microbiome, in addition to genetic predisposition, requiring additional layers of investigation to address the patient-specific disease mechanisms (Fig. 6). Most prominently, type 2 diabetes (T2D) is a global epidemic affecting over 400 million people and is a leading cause of morbidity and mortality (Menke et al., 2015). The prevalence of T2D is increasing as obesity and sedentary lifestyle are becoming more common in developing countries. The United States has the highest lifetime health care costs associated with T2D, reaching an estimated average of $283,000 per patient.

The primary cause for mortality in diabetic patients is cardiovascular disease (Haffner et al., 1998). T2D is associated with myocardial contractile dysfunction through accelerated atherosclerosis, diabetic cardiomyopathy, and sudden cardiac death. T2D is due to insulin resistance, an impaired response of the body to insulin despite hyperinsulinemia (Malik et al., 2004), as well as the failure of the pancreas to secrete adequate insulin for glucose homeostasis. Insulin resistance can precede the onset of T2D and is commonly referred to as prediabetes (Reaven, 2012).

Insulin resistance is associated with a metabolic syndrome that includes hypertension and dyslipidemia. Insulin resistance is often undiagnosed because it is difficult to test, but, like T2D, it is associated with an increased risk of cardiovascular disease (Reaven, 2012). In addition to requiring more insulin for glucose homeostasis, insulin resistance affects endothelial function. Nitric oxide release is impaired and is hypothesized to be a precursor of atherosclerosis (Arcaro et al., 2002; Kearney, 2013). Diabetes and insulin resistance are polygenic disease (Brown and Walker, 2016). iPSCs can serve as a renewable source of vascular tissue to study the mechanisms of insulin resistance and to find new personalized therapies that prevent cardiovascular disease in diabetic and insulin-resistant patients. For further details on the use of human iPSCs for pancreatic *β*-cell differentiation and regenerative therapy, we refer the readers to this review (Shahjalal et al., 2018).

T2D patients may also develop heart failure independent of these complications in a condition called diabetic cardiomyopathy, which was first documented in diabetic patients with adverse myocardial structural changes in the absence of hypertension and vascular dysfunction (Haffner et al., 1998). The exact mechanisms by which hyperglycemia induces diabetic cardiomyopathy are not fully understood, and current clinical management does not specifically address diabetes-induced heart failure (Jia et al., 2016). As such, resolving the contributing mechanisms of diabetic cardiomyopathy is a pressing goal of basic and translational research. iPSC-CMs offer an ideal platform to investigate the mechanisms of diabetic cardiomyopathy and develop new therapeutic targets, as studies have already shown that the in vitro exposure of iPSC-CMs to persistent insulin in the absence of glucose can mimic the physiologic diabetic cardiomyopathy conditions (Drawnel et al., 2014). The sodium-glucose transport protein 2 inhibitor, empagliflozin, was recently found to improve cardiovascular outcomes, including heart failure. This study showed that high glucose and insulin increased sodium-glucose transport protein 2 expression and treatment with empagliflozin ameliorated cardiac dysfunction (Ng et al., 2018). In addition, a self-organizing, human iPSC-derived three-dimensional blood vessel organoid model was developed to model structural and functional characteristics of diabetic microvasculopathy (Wimmer et al., 2019b). Exposure of the organoids to hyperglycemia and inflammatory cytokines to mimic the diabetic conditions resulted in vascular basement membrane thickening and splitting without increasing the vessel diameter, which was rescued when treated with *γ*-secretase inhibitor DAPT. Development of such iPSC-based organoid systems holds promise in advanced patient-specific disease modeling and drug screening and testing for diabetic cardiomyopathy and vasculopathies.

## X. Concluding Remarks

Cardiovascular drug discovery is enormously challenging due to factors such as the unavailability of cardiac cells, combinatorial etiology of cardiovascular diseases, and imperfect translation of animal studies to human subjects. Combined with the state-of-the-art tools in multiomics analysis (Wirka et al., 2018; Lau et al., 2019), the advent of iPSC technology has been pivotal in overcoming these limitations by providing a platform to better understand the patient-specific disease mechanisms and help to discover new personalized therapeutics.

However, a number of challenges in the use of iPSC technology for cardiovascular disease modeling and drug screening remains to be resolved. These include the generation of adult-like and tissue-specific subtypes of iPSC-derived cardiovascular cells as well as development of methods to introduce nongenetic etiological factors, all of which are currently under intensive investigation. Because the multifaceted pathophysiology of cardiovascular diseases involves multiple cell types in a number of organ systems, in vivo model validation is necessary to delineate the comprehensive disease phenotype, which should be performed concurrently to verify the findings from the iPSC-based in vitro studies. In addition, it will be necessary to generate functional and physiologic three-dimensional cardiac organoids and to develop high-throughput platforms utilizing heart and vessel mimics to enable effective and accurate drug screening and testing.

Notwithstanding these challenges, continuing advances have made the use of patient-specific iPSCs an indispensable and powerful tool in the discovery of effective therapeutics for all cardiovascular diseases, helping to realize the full potential of cardiovascular precision medicine.

## Abbreviations

BMP: bone morphogenetic protein
BMPR2: BMP receptor 2
CANTOS: Canakinumab Anti-Inflammatory Thrombosis Outcome Study
CM: cardiomyocyte
cTnI: cardiac troponin I
DCM: dilated cardiomyopathy
DEX: dexamethasone
DIC: doxorubicin-induced cardiotoxicity
EC: endothelial cell
ERK: extracellular signal-regulated kinase
ESC-CM: embryonic stem cell–derived CM
FDA: Food and Drug Administration
HCM: hypertrophic cardiomyopathy
hERG: human ether-à-gogo–related gene
iPSC: induced pluripotent stem cell
iPSC-CM: iPSC-derived CM
LDL: low-density lipoprotein
LMNA: lamin A/C
LVNC: left ventricular noncompaction
MESP1: mesoderm posterior protein 1
MHC: myosin heavy chain
MLC: myosin light chain
NKX2-5: NK2 homeobox 5
NRVM: neonatal rat ventricular myocyte
PAH: pulmonary arterial hypertension
PCL: poly-*ε*-caprolacton
PCSK9: proprotein convertase subtilisin/kexin type 9
PDGF: platelet-derived growth factor
SGLT2: sodium-glucose transport protein 2
ssTnI: slow skeletal troponin I
T2D: type 2 diabetes
T3: tri-iodothyronine
TGF: transforming growth factor
TKI: tyrosine kinase inhibitor
TNNT2: troponin T2, cardiac type
TOP2B: topoisomerase II-*β*
VEGF: vascular endothelial growth factor
vSMC: vascular smooth muscle cell
